# Prognostic value of combined MTV and ADC derived from baseline FDG PET/MRI in aggressive non-Hodgkins lymphoma

**DOI:** 10.1186/s12885-022-10194-2

**Published:** 2022-11-01

**Authors:** Trine Husby, Håkon Johansen, Trond Velde Bogsrud, Kari Vekseth Hustad, Birte Veslemøy Evensen, Ronald Boellaard, Guro F. Giskeødegård, Unn-Merete Fagerli, Live Eikenes

**Affiliations:** 1 Department of Circulation and Medical Imaging, Faculty of Medicine and Health Sciences, Norwegian University of Science and Technology, Postboks, 8905 Trondheim, Norway; 2 Department of Oncology, St. Olavs hospital, Trondheim University Hospital, Trondheim, Norway; 3 Department of Radiology and Nuclear Medicine, St. Olavs hospital, Trondheim University Hospital, Trondheim, Norway; 4 PET-Centre, University Hospital of North Norway, Tromsø, Norway; 5 PET-Centre, Aarhus University Hospital, Aarhus, Denmark; 6 Department of Nuclear Medicine and Molecular Imaging, University Medical Center Groningen, Groningen, The Netherlands; 7 Department of Radiology and Nuclear Medicine, Cancer Center Amsterdam, University Medical Centers Amsterdam, VUMC, Amsterdam, The Netherlands; 8 Department of Public Health and Nursing, Faculty of Medicine and Health Sciences, Norwegian University of Science and Technology, Trondheim, Norway; 9 Department of Clinical and Molecular Medicine, Faculty of Medicine and Health Sciences, Norwegian University of Science and Technology, Trondheim, Norway

**Keywords:** PET/MRI, Lymphoma, Metabolic tumor volume, Apparent diffusion coefficient

## Abstract

**Purpose:**

The aim of this prospective study was to investigate the prognostic value of metabolic tumor volume (MTV) and apparent diffusion coefficient (ADC) from baseline FDG PET/MRI compared to established clinical risk factors in terms of progression free survival (PFS) at 2 years in a cohort of diffuse large B-cell Lymphoma (DLBCL) and high-grade-B-cell lymphoma (HGBCL).

**Methods:**

Thirty-three patients and their baseline PET/MRI examinations were included. Images were read by two pairs of nuclear medicine physicians and radiologists for defining lymphoma lesions. MTV was computed on PET, and up to six lymphoma target lesions with restricted diffusion was defined for each PET/MRI examination. Minimum ADC (ADC_min_) and the corresponding mean ADC (ADC_mean_) from the target lesion with the lowest ADC_min_ were included in the analyses. For the combined PET/MRI parameters, the ratio between MTV and the target lesion with the lowest ADC_min_ (MTV/ADC_min)_ and the corresponding ADC_mean_ (MTV/ADC_mean_) was calculated for each patient. Clinical, histological, and PET/MRI parameters were compared between the treatment failure and treatment response group, while survival analyses for each variable was performed by using univariate Cox regression. In case of significant variables in the Cox regression analyses, Kaplan-Meier survival analyses with log-rank test was used to study the effect of the variables on PFS.

**Results:**

ECOC PS scale ≥2 (*p* = 0.05) and ADC_mean_ (*p* = 0.05) were significantly different between the treatment failure group (*n* = 6) and those with treatment response (*n* = 27). Survival analyses showed that ADC_mean_ was associated with PFS (*p* = 0.02, [HR 2.3 for 1 SD increase]), while combining MTV and ADC did not predict outcome. In addition, ECOG PS ≥2 (*p* = 0.01, [HR 13.3]) and histology of HGBCL (*p* = 0.02 [HR 7.6]) was significantly associated with PFS.

**Conclusions:**

ADC_mean_ derived from baseline MRI could be a prognostic imaging biomarker for DLBCL and HGBCL. Baseline staging with PET/MRI could therefore give supplementary prognostic information compared to today’s standard PET/CT.

## Introduction

Accurate baseline staging and clinical risk assessment scores are important to optimize treatment strategies in diffuse large B-cell Lymphoma (DLBCL) and high-grade-B-cell lymphoma (HGBCL). In addition, we need reliable prognostic imaging biomarkers to improve outcome for this patient population.

Functional imaging with ^18^F-Fluorodeoxyglucose (FDG) positron emission tomography (PET)- computed tomography (CT) is well established in baseline staging and treatment response for FDG-avid lymphomas. Magnetic resonance imaging (MRI) with its great soft tissue contrast is a radiation free alternative to CT. MRI has the advantage of adding functional imaging techniques like diffusion weighted imaging (DWI) to the standard morphological MR images, and studies has shown that hybrid FDG PET/MRI is a reliable alternative to PET/CT in lymphoma patients when it comes to baseline staging and response assessment [1–7].

A potential advantage of PET/MRI is the possibility of combining metabolic activity from PET with functional imaging from DWI as prognostic imaging biomarkers at baseline. Baseline metabolic tumor volume (MTV) measured on PET(/CT) is a promising prognostic imaging biomarker for DLBCL. Several studies have demonstrated that low MTV at baseline is associated with better progression free survival (PFS) and/or overall survival (OS) [8–10]. We have previously found good agreement between MTV from PET/CT and PET/MRI in patients with DLBCL and HGBCL [7]. DWI and its apparent diffusion coefficient (ADC) measures random motion of water molecules in tissues [11]. Low ADC has been found to be an independent unfavorable prognostic factor in primary central nervous system lymphoma (PCNSL) [12–14], while in a cohort of Hodgkins lymphoma, Punwani et al. showed that disease sites with an inadequate, interim treatment response had significantly higher pretreatment ADC [15].

By combining PET and MRI, a study in head and neck cancer [16] found that the ratio between MTV and ADC_mean_ was an independent prognostic factor for treatment failure. However, there is a lack of studies combining baseline ADC and MTV as possible prognostic parameters in systemic lymphoma. The aim of this prospective study was therefore to investigate the potential prognostic value of MTV and ADC derived from baseline PET/MRI compared to established clinical risk factors in terms of PFS at 2 years in a cohort of DLBCL and HGBCL.

## Materials and methods

### Study population

**Fig. 1:**
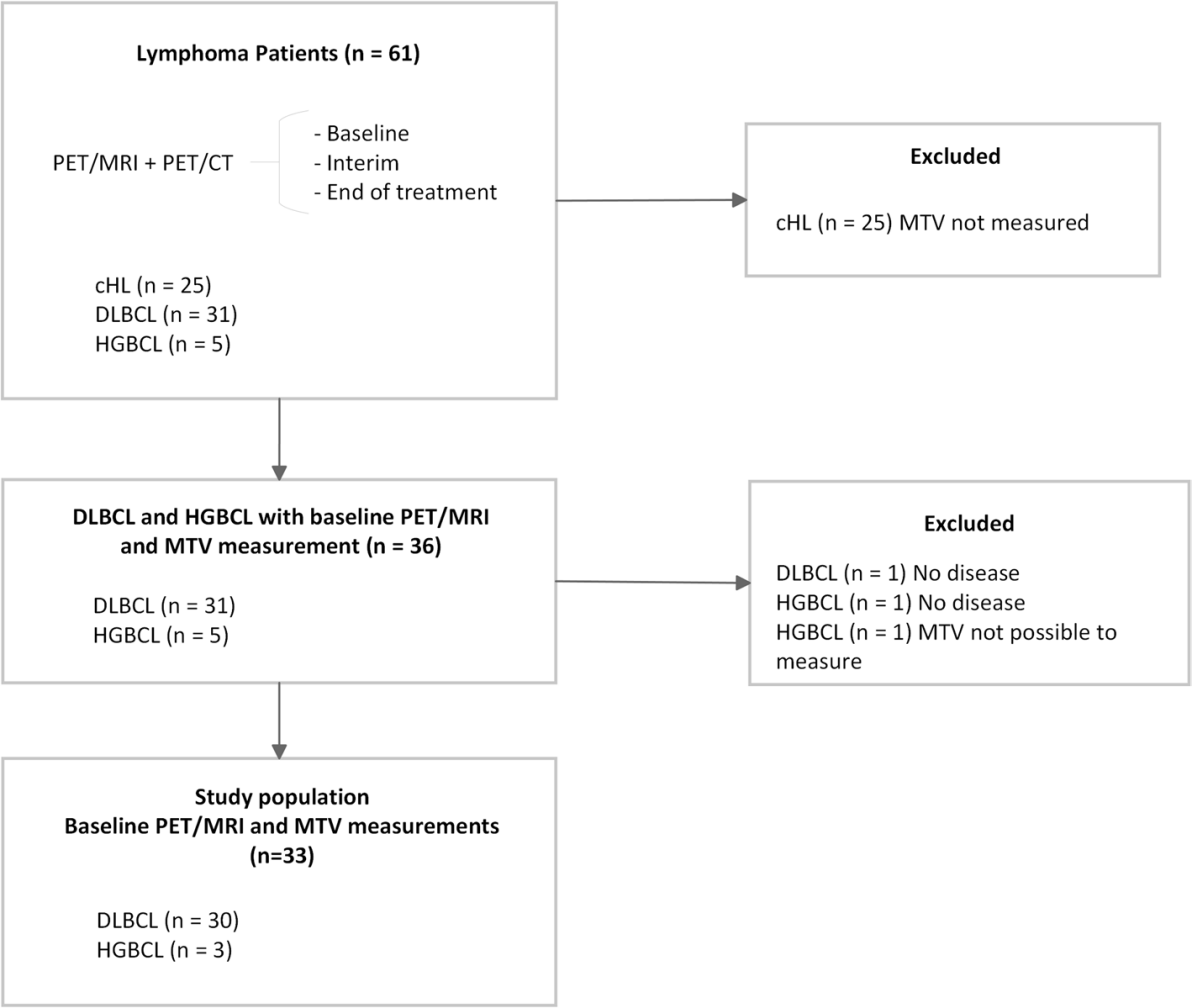
Flowchart of included patients in the study. Only baseline PET/MRI on aggressive non-Hodgkin lymphoma with measured metabolic tumor volume was included in this study. cHL = Classical Hodgkins lymphoma, DLBCL = Diffuse Large B-cell lymphoma, HGBCL = High-grade B-cell lymphoma, MTV = Metabolic tumor volume

Patients were enrolled from the lymphoma section at St. Olavs hospital, Trondheim University Hospital from June 2016 to February 2019. The study population were a subgroup drawn from a larger study [7] where 61 adult lymphoma patients with either classical Hodgkins lymphoma, DLBCL or HGBCL was scanned with PET/CT directly followed by PET/MRI at baseline and for response assessment during first line treatment. Only baseline PET/MRI data from the patients with DLBCL or HGBCL and those with MTV calculations was used in this current study, comprising a subgroup of 36 patients (Fig. 1). Two patients were not included in this analysis due to no detectable disease and one was excluded due to difficulty separating lymphoma tissue from bladder and kidney when computing MTV. A total of 33 patients and their baseline PET/MRI examinations were therefore included in the current study (Table 1). The study was approved by the Regional Committee for Ethics in Medical Research (REK-Midt #2014/1289). All participants gave written informed consent before participation.

**Table 1:** Study patient population. Baseline characteristics, treatment, and treatment response

Patient population	*N* = 33
Age, median	63
Age > 60	22
Gender	
Male	22
Female	11
Histology	
DLBCL	30
HGBCL	3
Number of extranodal sites	
≥ 2	10
Ann Arbor stage	
I-II	10
III-IV	23
Bulky tumor **≥**10 cm	18
Prognostic score, IPI	
IPS 0–3	26
High: IPS ≥ 4–5	7
Elevated LDH	15
B-symptoms	12
ECOG PS	
≥ 2	6
Systemic treatment	
R-CHOP/R-CHOEP	30
DA-EPOCH-R	2
GMALL-2002 + auto SCT	1
Consolidating radiation therapy	12
Response to treatment	
CMR	27
PD	4
Death during treatment	2

*DLBCL* Diffuse Large B-cell lymphoma, *HGBCL* High-grade B-cell lymphoma, *IPI* International Prognostic Index, *LDH* Lactate Dehydrogenase, *ECOG PS* Eastern Cooperative Oncology Group Performance Status, *R-CHOP/R-CHOEP* Rituximab, Cyclophosphamide, Doxorubicin, Vincristine, Prednisolone/Etoposide, *DA-EPOCH-R* Dose-adjusted Etoposide, Prednisolone, Vincristine, Cyclophosphamide, Doxorubicin and Rituximab GMALL-2002 = Blocks of cycles A,B and C containing Rituximab and intensive chemotherapy, *auto SCT* Autologous stem cell transplant, *CMR* complete metabolic response, *PD* progressive disease

### PET/MRI acquisition and image reconstruction

PET/MRI data was acquired on a Siemens Biograph mMR (Siemens Healthineers, Erlangen, Germany), 97 (median) minutes (range 87–150) after injection of FDG (4 MBq/kg). All patients fasted for at least 6 h before injection of FDG. None of the patients had hyperglycemia (10 mmol/L). Patients were imaged with their arms down in 5 bed positions covering the top of the skull to upper thighs, 5 min for each bed position. Simultaneous MRI was acquired with the following MRI sequences: Coronal T1 Dixon-VIBE, transversal diffusion weighted MRI (DWI) (b-values 50 and 800), transversal T2-HASTE and coronal T2-TIRM. Breath-hold imaging were used for bed positions 2–4, covering thorax and abdomen. Attenuation correction maps was calculated from the T1 Dixon-VIBE sequence, segmenting four tissue types (air, soft tissue, fat and lung) into predefined linear attenuation coefficients. PET image reconstruction was performed with iterative reconstruction (3D OSEM algorithm, 3 iterations, 21 subsets, 4 mm Gaussian filter) with point spread function (PSF), decay-, attenuation-, and scatter-correction and a 344 × 344 matrix.

### Image reading and staging

PET/MRI images were read by two pairs of nuclear medicine physicians (7 and 24 years of experience) and radiologists (13 and 14 years of experience) using a standardized reading protocol to identify lymphoma lesions. The PET and MR images were first interpreted separately by the nuclear medicine physicians and the radiologists, followed by a joint report for PET/MRI for each reading pair. The readers were blinded for clinical status. In case of disagreement between the two reading pairs, a final consensus was made by a third group consisting of a clinician with access to biopsy results, primary staging and follow up results and one radiologist from one of the two reading pairs and the nuclear medicine physician from the other reading pair. Standard clinical software, Advantage Server (GE Healthcare) and Syngo.Via (Simens Healthineers, Erlangen, Germany) were used for the PET and MRI reading, respectively.

For the PET reading, the Lugano Classification [17] criteria for staging were used. Diffuse uptake in the spleen without focal lesions had to be higher than 150% of SUV_max_ in the liver to be classified as diffusely involved [18].

For the MRI reading, a lymph node of > = 15 mm in largest diameter on axial sequences was defined as pathological for lymphoma involvement. Morphological criteria for splenic involvement were craniocaudal diameter more than 13 cm on coronal MRI or focal lesions. Bulky tumor was defined as > = 10 cm in largest diameter [19]. The reading of the different MRI sequences was performed simultaneously with no distinct order to combine morphological and structural information.

Anatomical staging in terms of extent of lymphoma disease with the modified Ann Arbor staging system [17] was performed separately by a lymphoma oncologist based on the joint PET/MRI report.

### MTV

MTV was computed separately by the two nuclear medicine physicians on PET from the baseline PET/MRI baseline examination using the research software ACCURATE, a semi-automatic software tool for quantitative analysis of PET images [20]. Initially, an automated analysis was done with fixed SUV-threshold of ≥4.0 and volume threshold ≥3 ml [21] before physiological uptake was excluded manually from the volume. Since intraclass correlation coefficient (ICC) showed excellent reliability (0,96) for the MTV measurements between the two readers on PET/MRI [7], we only used the MTV data from one of the readers in the analyses.

### ADC in target lesions

Based on DWI, T2-HASTE and PET images, one of the radiologists defined 1–6 (depending on stage and disease localization) FDG avid lymphoma target lesions with restricted diffusion for each PET/MRI examination. Structures that in normal state has restricted diffusion [22] was avoided if other lymphoma lesions were available for ADC measurements. Criteria for restricted diffusion on DWI was high signal on b50 (greater than surrounding muscle), persistent or increased signal on b800 and corresponding low signal on the ADC map [23]. The same radiologist measured the ADC of the target lesions in a manually defined ROI on the axial ADC map on the slice showing the maximum transversal diameter [24]. Necrotic, cystic, and vascular areas were avoided. The minimum ADC (ADC_min_) and the mean ADC (ADC_mean_) of the target lesions were reported. For each patient the ADC_min_ and the corresponding ADC_mean_ from the target lesion with the lowest ADC_min_ were included in the analyses.

### Combined MTV and ADC

The ratio between MTV and the target lesion with the lowest ADC_min_ (MTV/ADC_min)_ and the corresponding ADC_mean_ (MTV/ADC_mean_) were calculated for each patient.

### Clinical data

Well-established risk factors in terms of sex, age, bulky tumor, B-symptoms, performance status, histology, and Lactate Dehydrogenase (LDH) were recorded for the patients. The risk assessment score, International Prognostic Index score (IPI) 0–5, was used. Treatment regime in terms of type of immunochemotherapy and the use of consolidation radiation therapy or autologous stem cell transplant (auto-SCT) was recorded for each patient at end of treatment. All patients were staged with bone marrow biopsy in addition to either excisional biopsy or core needle biopsy of a systemic lymphoma lesion as part of standard clinical practice. The histological data was collected from a clinician with access to all the pathology reports.

### Response to treatment

Response to treatment was assessed based on clinical response assessment imaging (CT during treatment and PET/CT at end of treatment) and review of medical records. PET/CT is the gold standard for end of treatment response in aggressive non-Hodgkins lymphoma [17]. Based on this assessment, the patients were divided into two groups, treatment response or treatment failure group. The treatment response group were those who had a complete metabolic response on PET/CT at end of treatment. The treatment failure group were those who had progressive disease during treatment, biopsy confirmed residual metabolic disease at end of treatment, or suffered from a cancer related death before end of treatment.

### PFS

Twenty-four months PFS was calculated from the date of baseline PET/MRI imaging to the date of progression of disease, relapse or death from any cause. PFS was monitored by a clinician with access to follow up examinations every 3 months, imaging and biopsy results.

### Statistical analyses

All statistical analyses were performed using SPSS version 26.0. Proportions, range, and means were used for reporting descriptive statistics for the baseline and treatment response characteristics. Clinical, histological, and baseline PET/MRI parameters were compared between the treatment failure and treatment response group, using Fisher ´exact test for categorical variables or independent sample t-test for normally distributed continuous variables. Normal distribution of data was assessed by qq-plots and histograms. In the survival analyses, 24 months PFS was used as end point. The hazard ratio (HR) and 95% confidence interval (CI) were calculated for a one standard deviation (SD) increase in the level of each variable using univariate Cox regression. In case of significant PET/MRI variables in the Cox regression analyses, Kaplan-Meier survival analyses with log-rank test was used to study the effect of the variables on PFS. The median values of the PET/MRI parameters were used for cut off values. *P* values < 0.05 were considered statistically significant.

## Results

### Treatment and treatment response

Table 1 summarize the baseline characteristics, treatment regimens and treatment response of the patient population. Thirty patients with DLBCL (91%) received R-CHOP or R-CHOEP. Of the 3 patients with HGBCL (9%), 2 were treated with DA-EPOCH-R and 1 with GMALL-2002 regime including consolidation with auto SCT due to secondary CNS involvement. Twelve patients (36%) received consolidation radiation therapy. Twenty-seven patients (82%) achieved complete metabolic response at end of treatment response assessment and were therefore assigned to the treatment response group. Four patients (12%) had progressive disease during or at end of treatment and 2 patients (6%) died during treatment (cancer related death), thus 6 patients were assigned to the treatment failure group.

### Pre-treatment clinical and histological data

ECOC PS scale ≥2 was significantly different between the treatment failure group and those with treatment response (*p* = 0.05). However, no significant difference was found for the other risk factors in the treatment response group compared to the treatment failure group (Table 2).

**Table 2:** Comparison of clinical, histological, and PET/MRI parameters between the treatment failure and treatment response groups

Clinical and histological parameters	Treatment failure (*n* = 6)	Treatment response (*n* = 27)	*P* value
ECOG PS ≥2 n (%)	4 (67)	2 (7)	*.05*
HGBCL n (%)	2 (33)	1 (4)	.08
Age mean (range)	66.5 (50–75)	61.67 (33–82)	.41
Gender – male n (%)	4 (67)	18 (67)	.99
Number of extranodal sites ≥2 n (%)	3 (50)	9 (33)	.64
Ann Arbor stage III/IV n (%)	4 (67)	19 (70)	.99
Bulk n (%)	3 (50)	15 (56)	.99
IPI high ≥ 4–5 n (%)	2 (33)	6 (22)	.62
LDH elevated n (%)	4 (67)	16 (59)	.99
B-symptoms n (%)	3 (50)	9 (33)	.64
PET/MRI parameters			
ADC_mean_ (mm^2^/s) mean (range)	941 (472–1721)	710 (292–1126)	.05
ADC_min_ (mm^2^/s) mean (range)	466 (263–666)	357 (91–707)	*.20*
MTV (cm^3^) mean (range)	784 (155–2752)	527 (4–2474)	.38
MTV/ADC_mean_ mean (range)	0.91 (0.15–2.92)	0.71 (0.01–3.62)	.61
MTV/ADC_min_ mean (range)	1.68 (0.23–4.99)	1.64 (0.02–7.47)	.97

*ECOG PS* Eastern Cooperative Oncology Group Performance Status, *HGBCL* High-grade B-cell lymphoma, *IPI* International Prognostic Index, *LDH* Lactate Dehydrogenase, *ADC* Apparent diffusion coefficient, *MTV* Metabolic tumor volume

In the survival analyses the univariate Cox regression showed that ECOG PS ≥2 (*p* = 0.01, [HR 13.3]) and histology in terms of HGBCL (*p* = 0.02 [HR 7.6]) was significantly associated with treatment failure (Table 3). No statistically significant association with PFS was found for age, gender, number of extranodal sites ≥2, Ann Arbor stage I/II versus III/IV, bulk, IPI high ≥4–5, elevated LDH or B-symptoms (*p* > 0.05).

**Table 3:** Univarate Cox regression analyses for clinical, histological, and PET/MRI parameters at baseline

Prognostic factor	Hazards ratio (95% CI)	*P* value
ECOG PS ≥2	13.3 (2.4–75.2)	*.01*
HGBCL	7.6 (1.4–43.0)	*.02*
Age	1.0 (0.9–1.1)	.42
Gender	1.0 (0.2–5.6)	.97
Number of extranodal sites ≥2	1.8 (0.4–8.9)	.47
Ann Arbor stage III/IV	0.8 (0.2–4.8)	.87
Bulk	0.8 (0.7–3.9)	.77
IPI high ≥4–5	1.6 (0.3–9.0)	.56
LDH elevated	1.2 (0.2–6.7)	.81
B-symptoms	1.9 (0.4–9.6)	.42
ADC_mean_	2.3 (1.1–5.5)	*.02*
ADC_min_	1.8 (0.8–4.2)	.19
MTV	1.4 (0.7–2.7)	.34
MTV/ADC_mean_	1.8 (0.2–14.4)	.58
MTV/ADC_min_	1.0 (0.2–4.6)	.96

*ECOG PS* Eastern Cooperative Oncology Group Performance Status, *HGBCL* High-grade B-cell lymphoma, *IPI* International Prognostic Index, *LDH* Lactate Dehydrogenase, *ADC* Apparent diffusion coefficient, *MTV* Metabolic tumor volume

### Baseline MTV and ADC

When analyzing the difference in PET/MRI parameters between the treatment response and the treatment failure group, ADC_mean_ was the only parameter that was significantly different between the groups (*p* = 0.05) (Table 2). Although mean MTV levels were higher in the treatment failure than in the treatment response group, the difference was not statistically significant (*p* = 0.38). In addition, no significant difference was found for ADC_min_, MTV/ADC_min_ or MTV/ADC_mean_ between the two groups (*p* > 0.05).

**Fig. 2:**
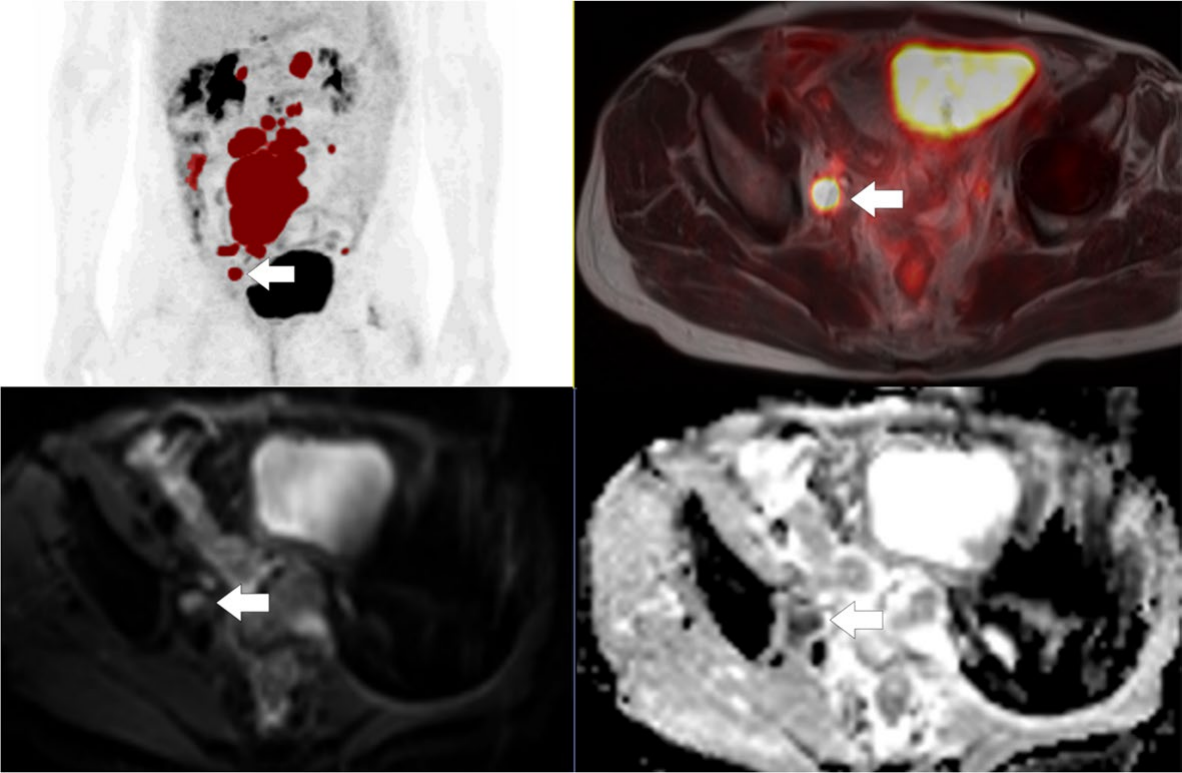
Baseline PET/MRI of a73-year-old male with Diffuse Large B-cell Lymphoma, stage IIAX, IPI 3 in progression at end of treatment scan. Metabolic tumor volume (MTV) was 375 cm^3^. The region of interest was a 16x11mm FDG avid target lesion in right pelvic/iliac region. Minimum apparent diffusion coefficient (ADC_min_) was 365 mm^2^/s and mean apparent diffusion coefficient (ADC_mean_) was 770 mm^2^/s. Upper left: PET MIP MTV. Upper right: Fused PET with T2-HASTE. Lower left: Diffusion weighted imaging (b800). Lower right: ADC-map

**Fig. 3:**
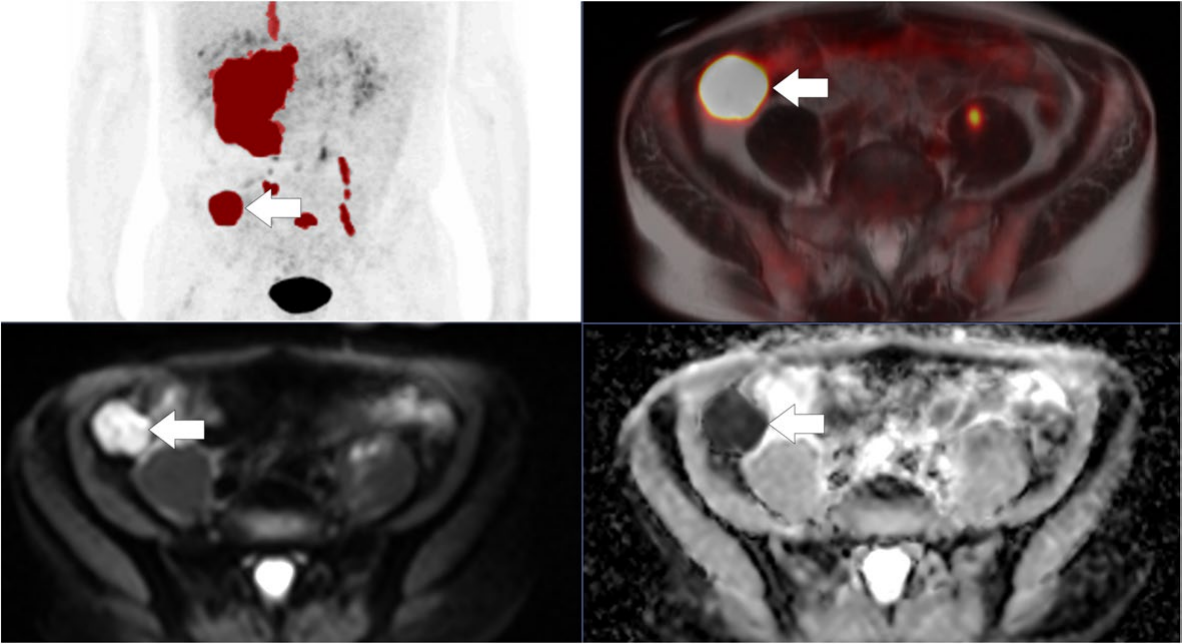
Baseline PET/MRI of a 64-year-old male with Diffuse Large B-cell Lymphoma, stage IVBX and IPI 4 in complete remission 2 years after baseline staging. Metabolic tumor volume was 563 cm^3^. The region of interest was a 30x33mm FDG avid target lesion in the right pelvic/iliac region. Minimum apparent diffusion coefficient (ADC_min_) was 165 mm^2^/s and mean apparent diffusion coefficient (ADC_mean_) was 545 mm^2^/s. Upper left: PET MIP MTV. Upper right: Fused PET with T2-HASTE. Lower left: Diffusion weighted imaging (b800). Lower right: ADC-map

**Fig. 4:**
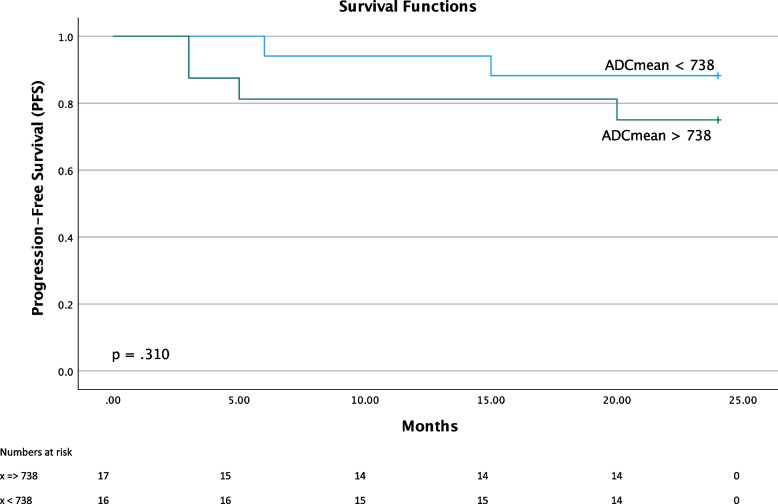
Kaplan-Meier curves for patients with high and low mean apparent diffusion coefficient (ADC_mean_) values. Progression-free survival is plotted against time (in months) for ADC_mean_ dichotomized to above or below the median value (median = 738 mm^2^/s)

Univariate Cox regression survival analyses showed that ADC_mean_ on a continuous scale was associated with PFS: patients with a low ADC_mean_ had a significantly better 2 years PFS than those with a high ADC_mean_ (*p* = 0.02, [HR 2.3 for 1 SD increase]) (Table 3). This is exemplified in two patients in the treatment failure and treatment response groups (Figs. 2 and 3). Although ADC_mean_ was associated with patient PFS in the univariate cox regression analysis, there was no significant difference when dividing the patients into two groups based on median value of ADC_mean_ in the Kaplan Meyer analysis (log rank *p* value = 0.31) (Fig. 4). No statistical significant association was found between the other PET/MRI parameters (ADC_min_, MTV, MTV/ADC_min_ or MTV/ADC_mean_) and outcome in terms of 2 year PFS (*P* > 0.05).

## Discussion

In this prospective study, we have investigated the prognostic value of biomarkers from baseline PET/MRI in a cohort of 33 patients with DLBCL and HGBCL. Our main findings were that ADC_mean_ was predictive of patient outcome, while combining PET data in terms of MTV with ADC did not add predictive value. To our knowledge, this is the first study to investigate the prognostic value of baseline ADC and combined imaging biomarkers such as the ratio between the MTV and ADC in systemic aggressive NHL in terms of PFS.

ADC values provide a quantitative index of water diffusion characteristics and may therefore reflect the histopathological condition of tissues and organs. We found that high ADC_mean,_ (> 738 mm^2^/s) at baseline was a significant, unfavorable prognostic factor. Two former studies have focused on the role of ADC in predicting outcome in systemic lymphoma, but the results are discordant. In line with our results, a study of 39 patients with HL found that disease sites with an inadequate treatment response had significantly higher pretreatment ADC [15], while a pilot study including 27 patients with HL and NHL found that patients with ADC > 752 mm^2^/s before treatment had a lower probability of progression [25]. The reason for this discrepancy is hard to postulate. One explanation could be that different tumor types have shown different associations between ADC and tumor cell count. However, a meta-analyses demonstrated that the correlation between ADC and cellularity in lymphomas is weak, suggesting that ADC cannot be used as a cellularity biomarker in this entity [26]. The role of other histopathological features like extracellular matrix, tumor cell size, nucleic areas and micro vessel density on ADC is still not fully understood and could cause the difference in the ADC-results in different lymphoma subtypes.

Even though there was a trend of higher MTV in the treatment failure group compared to the treatment response group, MTV was not significantly predicative of outcome in our cohort. These results contrast with several published studies which demonstrated that high MTV is a robust and unfavorable prognostic factor in DLBCL [8–10]. Compared to these studies we have a larger fraction of limited disease (stage I and II), which could explain the negative result in the current study. The relatively large time interval between FDG injection and PET/MR acquisition start (87–150 min) in our patient cohort could also have an impact on the results. We have previously demonstrated a slight increase in SUV_max_ from PET/CT to PET/MR in the same lymphoma cohort, which could be related to the prolonged uptake time on PET/MR (97 min) versus PET/CT (60 min) [7]. However, in the same study MTV was slightly higher on PET/CT compared to PET/MR, making it difficult to postulate what effect the uptake time has on MTV. We also found excellent reliability (ICC = 0.99) between MTV from the two modalities, demonstrating that MTV from PET/MR is a robust measure regardless of the differences in uptake time.

Several of the well-established pre-treatment clinical risk factors for aggressive NHL like the presence of bulk, high LDH and stage (Ann Arbor) often reflects tumor burden and therefore also high MTV. Since neither of these were predictive of outcome in our patient population, this may further explain the lack of MTV as a significant prognostic factor in the current study. This may also have influenced the negative results of the combined prognostic imaging biomarkers from PET/MRI like the ratio between the MTV and ADC (MTV/ADC_min_ and MTV/ADC_mean_) in our study. The lack of significant results for both the clinical risk-factors and MTV could also be explained by the small sample size in our study. In the absence of other PET/MRI lymphoma studies focusing on the role of combined PET and MRI prognostic biomarkers, we must be careful to draw any firm conclusions based on our negative results. Therefore, large-scale studies are required to validate these results.

Our study also has limitations in terms of few events. Two years PFS is shown to be is a robust endpoint for newly diagnosed DLBCL treated with standard of care immunochemotherapy. Maurer and colleagues showed in two large cohorts that patients who are event-free at 24 months after diagnoses (roughly 70%) had an OS equivalent to the general population [27]. Despite our small cohort, 82% of the patients was in complete remission at 24 months, and the patient population in the current study is therefore quite representative. Furthermore, ADC was only measured in six or less FDG avid lymphoma lesions in each patient with extended disease and not in all the lymphoma lesions. It is therefore uncertain whether the lowest ADC_min_ was measured, which would affect the corresponding ADC_mean_ for the patients with large tumor volume.

To conclude, this study demonstrated that ADC_mean_ measured at baseline could be a prognostic imaging biomarker for DLBCL and HGBCL. Baseline staging with PET/MRI could therefore give supplementary prognostic information compared to PET/CT.

## Data Availability

The datasets generated and/or analysed during the current study are not publicly available due to the European Union General Data Protection Regulations (GDPR), but are available from the corresponding author on reasonable request.
